# Metabolic Engineering of Shikimic Acid-Producing *Corynebacterium glutamicum* From Glucose and Cellobiose Retaining Its Phosphotransferase System Function and Pyruvate Kinase Activities

**DOI:** 10.3389/fbioe.2020.569406

**Published:** 2020-09-10

**Authors:** Naoki Sato, Mayumi Kishida, Mariko Nakano, Yuuki Hirata, Tsutomu Tanaka

**Affiliations:** Department of Chemical Science and Engineering, Graduate School of Engineering, Kobe University, Kobe, Japan

**Keywords:** shikimic acid, shikimate pathway, *Corynebacterium glutamicum*, metabolic engineering, beta-glucosidase, cellobiose

## Abstract

The production of aromatic compounds by microbial production is a promising and sustainable approach for producing biomolecules for various applications. We describe the metabolic engineering of *Corynebacterium glutamicum* to increase its production of shikimic acid. Shikimic acid and its precursor-consuming pathways were blocked by the deletion of the shikimate kinase, 3-dehydroshikimate dehydratase, shikimate dehydratase, and dihydroxyacetone phosphate phosphatase genes. Plasmid-based expression of shikimate pathway genes revealed that 3-deoxy-D-arabino-heptulosonate 7-phosphate (DAHP) synthase, encoded by *aroG*, and DHQ synthase, encoded by *aroB*, are key enzymes for shikimic acid production in *C. glutamicum*. We constructed a *C. glutamicum* strain with *aroG, aroB* and *aroE3* integrated. This strain produced 13.1 g/L of shikimic acid from 50 g/L of glucose, a yield of 0.26 g-shikimic acid/g-glucose, and retained both its phosphotransferase system and its pyruvate kinase activity. We also endowed β-glucosidase secreting ability to this strain. When cellobiose was used as a carbon source, the strain produced shikimic acid at 13.8 g/L with the yield of 0.25 g-shikimic acid/g-glucose (1 g of cellobiose corresponds to 1.1 g of glucose). These results demonstrate the feasibility of producing shikimic acid and its derivatives using an engineered *C. glutamicum* strain from cellobiose as well as glucose.

## Introduction

Shikimic acid is a valuable hydroaromatic compound. It is a key metabolic intermediate in the shikimate pathway, a common route for the biosynthesis of a range of aromatic compounds. Shikimic acid possesses a highly functionalized six-carbon ring with three asymmetric centers, and is therefore, a useful precursor for the synthesis of products such as pharmaceuticals, antibiotics, antithrombotic agents, and vitamins (Rawat et al., 2013; Martinez et al., 2015; Candeias et al., 2018; Kogure and Inui, 2018). This compound has been used as a starting material for the industrial synthesis of oseltamivir phosphate, otherwise known as the anti-influenza drug Tamiflu (Díaz Quiroz et al., 2014). Shikimic acid is also an important starting material for the synthesis of anticancer drugs such as (+)-zeylenone (Liu et al., 2004). Current industrial production of shikimic acid primarily involves its extraction from the seeds of the Chinese star anise plant (almost 80% or more), *Illicium verum*, and subsequent chemical synthesis (Bochkov et al., 2012; Ghosh et al., 2012; Rawat et al., 2013). However, these processes are complex and incur problems such as limitations of raw material and high costs.

The production of biomolecules using microorganisms has recently attracted considerable attention because of the potential for relatively rapid, cost-effective, low-pollution production of industrially important compounds. Shikimic acid is of particular interest due to its importance in a range of industrial applications (Rawat et al., 2013; Martinez et al., 2015; Noda and Kondo, 2017). Microbial production of shikimic acid has several advantages: low-cost renewable feedstocks are readily available in large quantities; the system is environmentally friendly; and high yields can be obtained. Shikimic acid is produced via the shikimate pathway, which is ubiquitous in bacteria, plants, and fungi. This pathway begins with the condensation of phosphoenolpyruvate (PEP) and D-erythrose 4-phosphate (E4P), catalyzed by 3-deoxy-D-arabino-heptulosonate-7-phosphate (DAHP) synthetase. DAHP is converted to 3-dehydroquinate (DHQ) by DHQ synthase. DHS synthase catalyzes the next step to produce 3-dehydroshikimate (DHS), which is then converted to shikimic acid by shikimate dehydrogenase. Metabolic engineering for the production of useful compounds derived from the shikimate pathway, including shikimic acid, has been extensively studied (Jiang and Zhang, 2016; Lee and Wendisch, 2017; Noda and Kondo, 2017; Averesch and Krömer, 2018; Huccetogullari et al., 2019; Braga and Faria, 2020), primarily using *Escherichia coli* as the host strain. A typical strategy for shikimic acid production involves the elimination of the carbon flow from shikimate to chorismite by deletion of the shikimate kinase gene and the reinforcing of carbon flux from glycolysis into the shikimate pathway by overexpression of the shikimate pathway enzymes, including a feedback-resistant DAHP synthase (Gu et al., 2017). Improvement of PEP and E4P availability is another key issue for shikimic acid production. Deletion of pyruvate kinase, inactivation of the phosphotransferase system (PTS) and the redirection of carbon flux from pyruvate to PEP by PEP synthase expression have all been utilized (Chandran et al., 2003). Combining these metabolic approaches, Rodriguez et al. (2013) engineered a shikimic acid-producing *E. coli* strain, which achieved production of 43 g/L of shikimic acid at a yield of 43% (mol/mol) from glucose in a batch fermenter. However, the inactivation of PTS or pyruvate kinase often causes growth defects, and a laboratory-based adaptive evolution process is required to recover cell growth (Flores et al., 2005, 2007). Introduction of alternative glucose transporter Glf from *Zymomonas mobilis* with an additional copy of glucokinase from *Z. mobilis* could recover cell growth without applying an adaptive evolution experiment (Chandran et al., 2003). Another concern about pyruvate kinase and/or PTS deletion is the shortage of pyruvate supply. For the synthesis of various plant polyphenols such as flavonoids and stilbenoids, pyruvate and acetyl-CoA are important precursor as well as chorismic acid followed by shikimic acid synthesis (Kogure and Inui, 2018).

*Corynebacterium glutamicum* is a Gram-positive non-pathogenic bacterium, which is already widely used for the industrial production of amino acids such as L-glutamate and L-lysine (Hirasawa and Wachi, 2017; Ikeda, 2017; Baritugo et al., 2018; Becker et al., 2018; Cheng et al., 2018; D’Este et al., 2018; Pérez-García and Wendisch, 2018; Wendisch et al., 2018), and aromatic amino acids such as L-phenylalanine and L-tyrosine (Ikeda, 2006; Zhang C. et al., 2015). This microbe is one of the most promising microbial chassis for the bioproduction of a range of chemicals and fuels, including alcohols, diamines, and organic acids. Kogure et al. (2016) achieved a production of 141 g/L of shikimic acid from glucose using the *C. glutamicum* R strain using growth-arrested cell reactions in fed-batch fermentation. These researchers induced the expression of a heterologous gene encoding a feedback-resistant form of DAHP from *E. coli* (Ger et al., 1994), as well as shikimate pathway-related genes. In the engineered strain, PTS and the PEP-independent glucose uptake system were also inactivated. Another study described the production of an engineered *C. glutamicum* strain derived from strain ATCC13032 using CRISPRi system-mediated transcriptional control. This strain produced 7.76 g/L shikimic acid from sucrose in flask cultivation (Zhang et al., 2016). Zhang et al. constructed an engineered strain using ribosome binding site libraries (Zhang C. et al., 2015; Zhao et al., 2020) or the introduction of the archaeal shikimate pathway (Zhang et al., 2015a). The strains produced shikimic acid at 11.3 and 23.8 g/L, respectively, using sucrose as a carbon source.

Lignocellulosic biomass has attracted much attentions as a promising feedstock because it is renewable, inexpensive and abundant (Bhatia et al., 2020; Zhao et al., 2020). Bio-fuels and chemicals produced from lignocellulosic biomass has a potential to develop the sustainable economic growth. However, most microorganisms cannot directly utilize lignocellulosic biomass, and degradation of this material requires expensive and complex steps. To convert cellulose into monomeric sugars such as glucose, enzymetic saccharification procedures involving endoglucanase, exglucanase and beta-glucosidase (BGL) have been required (Khare et al., 2015). After cleaving the cellulose chain in the middle by endoglucanase, then cellobiohydrolases release cellobiose units from the end of the cleaved cellulose. Finally, BGL hydrolyze cellobiose and cello-oligosaccharides to glucose (Davies et al., 2005). The cellobiose and glucose causes product inhibition of BGL activity. To overcome this problem, excess amount of BGL is added to cellulase mediated hydrolysis (Van Dyk and Pletschke, 2012) or simultaneous saccharification and fermentation (SSF) processes (Liu et al., 2019). Other inhibitors such as tannic, gallic, vanillin were able to cause 20–80% BGL inhibition (Ximenes et al., 2011). It was found that amounts of disaccharides existed in the hydrolyzate, such as cellobiose (Li et al., 2010). Although shikimic acid production from the mixture of glucose, xylose and arabinose were carried out (Kogure et al., 2016), shikimic acid production using cellobiose or cello-oligosaccharides as a carbon source have not yet been used.

In this study, we constructed a high-producing shikimic acid *C. glutamicum* strain by rational metabolic engineering approaches without PTS inactivation, pyruvate kinase disruption, or the introduction of feedback-resistant variants of DAHP synthase. We simply disrupted shikimic acid and its precursor-consuming genes and introduced only endogenous genes related to the shikimate pathway. Our engineered strain produced 13.8 g/L of shikimic acid, a yield of 0.25 (g-shikimic acid/g-glucose). Further, we demonstrated successful shikimic acid production using cellobiose as the sole carbon source. The titer of shikimic acid production was 13.8 g/L with the same yield of glucose.

## Materials and Methods

### Bacterial Strains, Media, and Cultivation Conditions

All bacterial strains and plasmids used in this study are listed in Table 1. *C. glutamicum* ATCC13032 and its recombinants were cultivated aerobically at 30°C in Brain Heart Infusion (BHI) medium or defined CGXII minimal medium containing 50 g/L of glucose or cellobiose and 100 mg/L of aromatic amino acids. CGXIIY medium (CGXII medium containing 4 g/L of yeast extract) was used for shikimic acid production. *E. coli* NovaBlue, which was used for recombinant DNA experiments, was routinely cultivated in Luria–Bertani medium (10 g/L peptone, 5 g/L yeast extract, and 10 g/L NaCl) at 37°C. Kanamycin (25 μg/mL for *C. glutamicum* strains and 50 μg/mL for *E. coli*) was added when required.

**TABLE 1:** Bacterial strains and plasmids used in this study.

**Strains or plasmids**	**Genotype**	**Source or reference**
*Escherichia coli*		
Nova Blue	*endA1 hsdR17(rK12-mK12^+^) supE44 thi-I gyrA96 relA1 lac recA1/F’ [proAB^+^ lacIq Z*Δ*M15:Tn10(Tet r)];* used for gene cloning.	Novagen
SCS110	*rpsL (Str1) thr leu endA thi-l lacY galK galT ara tonA tsx dam dcm supE44*Δ*(lac-proAB) [F’ traD36 proAB laclqZ*Δ*M15*]	STRATAGENE
*Corynebacterium glutamicum*		
ATCC13032	Wild-type strain	ATCC
SA-1	ATCC13032 + ΔcglMRR (cg1996-cg1998), Δ*qsuD*,Δ*qsuB*,Δ*nagD*,Δ*aroK*	This study
SA-2	SA-1 + *gnd^ S361*F*^*	This study
SA-3	SA-2 + *aroK:P_*H*__36_-aroG*	This study
SA-4	SA-2 + *aroK:P_*H*__36_-aroB*	This study
SA-5	SA-2 + *aroG^*gtg*→*atg*^*,Δ*cg2392*	This study
SA-6	SA-5 + *aroK:P_*H*__36_-aroB*	This study
SA-7	SA-5 + *aroK:P_*H*__36_-aroB, pta:P_*H*__36_-aroG*	This study
SA-8	SA-7 + Δ*ldh*	This study
SA-9	SA-7 + *ldh:P_*H*__36_-aroE1*	This study
SA-10	SA-7 + *ldh:P_*H*__36_-aroE3*	This study
Plasmids		
pCCS	*E. coli–C. glutamicum shuttle vector for control, KmR*	Matsuura et al., 2019
pCC-H36-cgR0949-Tfu0937	pCCS derivative carrying sequences encoding the CgR0949 secretion signal fused to *T. fusca* Tfu0937 under the control of the H36 promoter	Matsuura et al., 2019
pCC-P_*H*__36_-aroG	pCCS containing *aroG* under the control of the H36 promoter	This study
pCC-P_*H*__36_-aroB	pCCS containing *aroB* under the control of the H36 promoter	This study
pCC-P_*H*__36_-qsuC	pCCS containing *qsuC* under the control of the H36 promoter	This study
pCC-P_*H*__36_-aroE1	pCCS containing *aroE1* under the control of the H36 promoter	This study
pCC-P_*H*__36_-aroE3	pCCS containing *aroE3* under the control of the H36 promoter	This study
pCC-P_*H*__36_-tal-tkt	pCCS containing *tal* and *tkt* (cg1774-cg1776, NCgl1512-NCgl1513) under the control of the H36 promoter	This study
pCC-P_*H*__36_-pck	pCCS containing *pck* under the control of the H36 promoter	This study
pK18mobsacB	sacB, lacZ, Km^*R*^, MCS, mobilizable vector, enables selection/counter-selection for integration/excision in *C. glutamicum*	ATCC
pK18-gnd^*S*361*F*^	pK18mobsacB derivative for substitution of *gnd^*S*361*F*^*	This study
pK18-ΔqsuD	pK18mobsacB derivative for *qsuD* deletion	This study
pK18-ΔqsuB	pK18mobsacB derivative for *qsuB* deletion	This study
pK18-ΔnagD	pK18mobsacB derivative for *nagD* deletion	This study
pK18-ΔaroK	pK18mobsacB derivative for *aroK* deletion	This study
pK18-Δldh	pK18mobsacB derivative for *ldh* deletion	This study
pK18-ΔaroK-P_*H*__36_aroB	pK18mobsacB derivative for insertion of *aroB* expression cassette under the control of H36 promoter into *aroK* locus	This study
pK18-Δpta-P_*H*__36_aroG	pK18mobsacB derivative for insertion of *aroG* expression cassette under the control of H36 promoter into *pta* locus	This study
pK18-Δldh-P_*H*__36_aroE1	pK18mobsacB derivative for insertion of *aroE1* expression cassette under the control of H36 promoter into *ldh* locus	This study
pK18-Δldh-P_*H*__36_aroE3	pK18mobsacB derivative for insertion of *aroE3* expression cassette under the control of H36 promoter into *ldh* locus	This study

### Construction of Plasmids and Strains

The strains and plasmids used in this study are summarized in Table 1. All DNA oligonucleotides used in this study are listed in Supplementary Table S1. Gene deletion or substitution plasmids were constructed as follows.

For the *aroK* deletion, the upstream and downstream regions were amplified from *C. glutamicum* strain ATCC 13032 by PCR using the primer pairs ΔaroK_up_fw/ΔaroK_up_rv, and ΔaroK_down_fw/ΔaroK_down_rv, respectively. The two fragments were conjugated by overlap PCR using the primer pair ΔaroK_up_fw/ΔaroK_down_rv, and the resulting fragment was ligated into pK18mobsacB digested using *Eco*RI/*Hin*dIII. Other plasmids for gene deletion (Δ*qsuD*, Δ*qsuB*, Δ*nagD*, and Δ*cg2392*) and for point mutation (gnd S361F) were constructed similarly.

We constructed an *aroG*-expressing plasmid under the control of the H36 promoter as follows. A gene fragment encoding *aroG* was amplified by PCR from *C. glutamicum* ATCC13032 using the primer pairs H36_aroG_fw and AroG_re. The fragment was ligated into plasmid pCC-H36-ldcC digested with *Bam*HI/*Xho*I. The resultant plasmid was named pCC-H36-aroG. Other plasmids expressing *aroB, qsuC, aroE1* and *aroE3* were constructed similarly.

For *aroG* integration into the Δ*aroK* region, the upstream and downstream regions of Δ*aroK* were amplified by PCR using the primer pairs aroK up fw/aroK up rv and aroK down fw/aroK down rv. An *aroG*-expressing cassette with the H36 promoter and rrnB T1T2 terminator was amplified by PCR using pCC-H36-aroG as a template with the primer pairs aroK-H36_fw/aroK_term_re. The three fragments were conjugated by overlap PCR using the primer pair aroK up fw and aroK down rv, and the resulting fragment was ligated into pK18mobsacB digested with *Eco*RI/*Bam*HI. Other plasmids for genome integration (Δ*aroK*:*aroB*, Δ*pta*:*aroG*,Δ*ldh*:*aroE1 and*Δ*ldh*:*aroE3*) were constructed similarly.

All gene deletions, mutations, and integrations of the *C. glutamicum* genome were introduced by homologous recombination and two-step selection using kanamycin resistance to select for plasmid integration and the *sacB* system to counterselect for plasmid excision.

### Transformation of *C. glutamicum*

*C. glutamicum* was cultured overnight in 5 mL of BHI medium at 30°C. A further 400 μL of seed culture was inoculated into 50 mL of BHI medium. After incubation at 30°C until OD_600_ reached 0.5, the cell suspension was centrifuged at 4,000 g for 5 min and washed three times with 5mL of 15% (v/v) glycerol before resuspension in 0.5 mL of 15% glycerol. Transformation of *C. glutamicum* was performed by electroporation with a 2.5-kV, 200-Ω, 25-μF electric pulse in a 0.2-cm cuvette using a Gene Pulser (Bio-Rad Laboratories, Hercules, CA).

### Conditions for Shikimic Acid Production by *C. glutamicum*

A single colony was used as the inoculum for preculture (5 mL BHI medium in test tubes) and was incubated overnight at 30°C with shaking at 220 rpm. Cells were collected by centrifugation at 4,000 g for 2 min and resuspended in 1 mL of CGXII medium supplemented with 100 mg/L of L-phenylalanine, 100 mg/L of L-tyrosine, 100 mg/L of L-tryptophan, and 100 mg/L of p-aminobenzoate. The suspension (400 μL) was inoculated into 5 ml of CGXII medium or CGXIIY medium containing 50 g/L of glucose and incubated at 30°C with shaking at 220 rpm 72 h.

### Analysis of Substrates and Products

Cell growth was determined by measuring the OD600 nm on a UVmini-1240 spectrophotometer (Shimadzu Corporation, Kyoto, Japan). Glucose concentration was analyzed using a Prominence high-performance liquid chromatography (HPLC) system (Shimadzu) equipped with a Shodex SUGAR KS-801 column (6 μm, 300 × 8.0 mm L × I.D., Shodex). The column was maintained at 50°C, and water was used as the mobile phase at a flow rate of 0.8 ml/min. The HPLC profile was monitored using a refractive index detector. The concentration of shikimic acid was determined using HPLC equipped with COSMOSIL PBr column (5 μm, 4.6 mm × 250 mm, I.D. × L, Nacalai Tesque). A 98:2 mixture of 0.2% phosphoric acid and methanol was used as the mobile phase at a flow rate of 1.0 ml/min, and the column was maintained at 40°C. The HPLC profile was monitored using a UV-VIS detector at 240 nm.

### Quantification of NADPH/NADP and NADH/NAD Ratio

Preparation of samples for the analysis of NADPH and NADH was conducted as follows. Precultured *C. glutamicum* was inoculated into 5 ml of CGXIIY medium containing 50 g/L of glucose and incubated at 30°C with shaking at 220 rpm for 24 h. The extraction buffer (methanol: chloroform = 7:3) was added to cells and incubated at 1,000 rpm at 4°C overnight. The supernatant was centrifuged at 13,000 g for 5 min and evaporated. The evaporated samples were analyzed using the Enzychrom NADP + /NADPH Assay Kit ECNP-100 (BioAssay Systems, Hayward, United States) and Enzychrom NAD + /NADH Assay Kit E2ND-100 (BioAssay Systems, Hayward, United States) according to the manufacturer’s procedures.

### Quantification of the Transcriptional Level of mRNA Using Real-Time qPCR

The transcriptional expression of *aroB*, *aroG*, and *ldh* in SA-2, SA-5, and SA-7 was quantified using real-time qPCR. Total RNA was isolated after 24 h of cultivation in CGXIIY medium using a NucleoSpin RNA column (Takara Bio, Shiga, Japan) according to the manufacturer’s protocol.

Quantitative real-time qPCR was performed using a LightCycler^®^ 96 System (Roche Molecular Systems, Inc., CA, United States) with RNA-direct^TM^ Real-time PCR Master Mix (TOYOBO). The primer pairs used are listed in Supplementary Table 1. The normalized transcriptional level of each mRNA was calculated using the relative quantification method using the cg3177 (Ncgl2772) gene as the housekeeping gene.

### Statistical Analysis

Analysis of variance (ANOVA) was conducted with Honestly Significant Difference (HSD) test by EZR software (Kanda, 2013). The data of shikimic acid are average of 3 biological replicates with error bars representing standard deviation and asterisk indicates significant difference (*p* < 0.05) from control strain.

## Results and Discussion

### Construction of Shikimic Acid-Producing *C. glutamicum*

**FIGURE 1:**
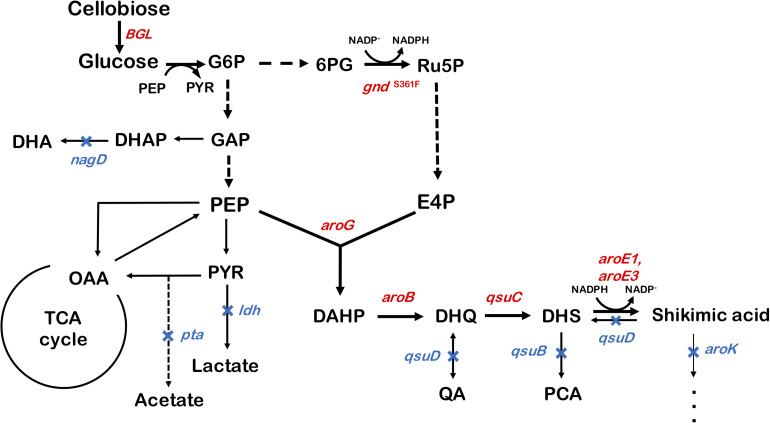
Metabolic engineering of *Corynebacterium glutamicum* for shikimic acid production. The blue X indicates deletion of the *nagD, qsuD, qsuB, qsuD, aroK*, and *pta* genes. Genes involving shikimic acid synthesis pathways are indicated in red. G6P, glucose-6-phosphate; GAP, glyceraldehyde-3-phosphate; DHAP, 1,3-dihydroxyacetone phosphate; DHA, 1,3-dihydroxyacetone; PEP, phosphoenolpyruvate; PYR, pyruvate; OAA, oxaloacetate; Ru5P, ribulose-5-phosphate; E4P, erythrose 4-phosphate; DAHP, 3-deoxy-D-arabinoheptulosonate-7-phosphate; DHQ, 3-de-hydroquinate; DHS, 3-dehydroshikimate; PCA, protocatechuate; *aroG*, DAHP synthase; *aroB*, DHQ synthase; *aroD*, DHQ dehydratase; *aroE1*, *aroE3*, shikimate dehydrogenase; *aroK*, shikimate kinase; *qsuB*, DHS dehydratase; *qsuD*, QA/shikimate dehydrogenase; *gnd*, 6-phosphogluconate dehydrogenase; *BGL*, beta-glucosidase from *Thermobifida fusca* YX.

**FIGURE 2:**
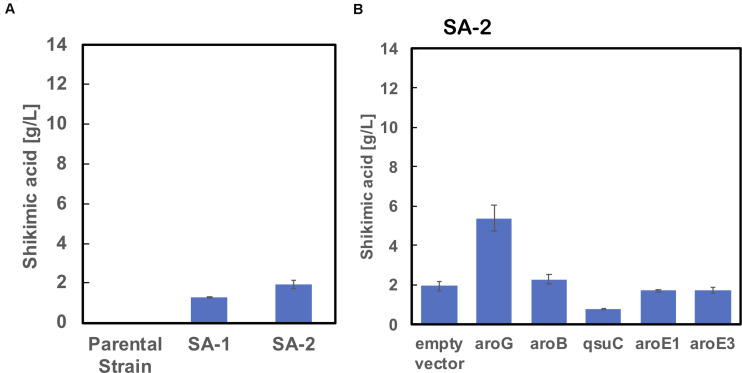
**(A)** Shikimic acid production titer in strains SA-1 and SA-2 harboring empty vectors. **(B)** SA production titer in SA-2 harboring each individual shikimate pathway gene expression plasmid. CGXII medium containing 50 g/L of glucose was used as the sole carbon source. The data are presented as the average of three independent experiments, and error bars indicate the standard deviation.

We used ATCC13032 ΔMRR (a partially prophage-free variant) as the parent strain for constructing shikimic acid-producing *C. glutamicum* (Bott et al., 2013). We disrupted four genes encoding shikimate kinase *aroK* (cg1828, NCgl1560); DHS dehydratase *qsuB* (cg0502, NCgl0407); QA/SA dehydratase *qsuD* (cg0504, NCgl0409); and DHAP phosphatase *nagD* (cg2474, NCgl2175), leading to the accumulation of shikimic acid (Figure 1). The strain, called SA-1 (D*aroK*, D*qsuB*, D*qsuD*, and *DnagD*), exhibited auxotrophy for aromatic amino acids and p-aminobenzoate. Therefore, small amounts of L-phenylalanine, L-tyrosine, L-tryptophan, and p-aminobenzoate (100 mg/L) had to be added to the minimal medium for the strain to grow. No shikimic acid was detected in the parental strain, but SA-1 produced 1.3 ± 0.04 g/L of shikimic acid in CGXII medium containing 50 g/L of glucose after 72 h of cultivation (Figure 2A). Ohnishi et al. reported that the point mutation Ser361Phe of the 6-phosphogluconate dehydrogenase gene (gnd; cg1643, NCgl1396) increased L-lysine production due to enhancement of NADPH supply (Ohnishi et al., 2005). Shikimate dehydrogenase *aroE* requires NADPH as a cofactor for its enzymatic activity, so we introduced this point mutation into SA-1 by allelic replacement. The resulting strain SA-2 produced 1.9 ± 0.23 g/L of shikimic acid after 72 h, an increase of approximately 40% over that of SA-1 (Figure 2A).

### Overexpression of Shikimate Pathway Genes in Strain SA-2 Using a Plasmid-Based Expression Method

To improve shikimic acid production, each gene of the shikimate pathway was overexpressed under the control of the strong synthetic constitutive promoter P_H__36_ (Yim et al., 2013). This promoter has been used to produce recombinant single-chain variable fragment (Yim et al., 2014), gamma-aminobutyrate (Choi et al., 2015), and 1,5-diaminopentane (Matsuura et al., 2019; Kim et al., 2020). Key genes located on the *C. glutamicum* chromosome are DAHP synthase, encoded by *aroG* (cg2391, NCgl2098); DHQ synthase, encoded by *aroB* (cg1827, NCgl1559); DHS synthase, encoded by *qsuC* (cg0503, NCgl0408); shikimate dehydrogenase, encoded by *aroE1* (cg1283, NCgl1087); and *aroE3* (cg1835, NCgl1567). Although *C. glutamicum* carries the DAHP synthase encoded by *aroF*, its contribution to the shikimate pathway is less than that of *aroG* (Liu et al., 2008), and we did not select it as a candidate. Individual plasmids were created for the overexpression of *aroG*, *aroB*, *qsuC*, *aroE1*, and *aroE3*, under the control of the strong constitutive promoter P_H__36_. Each plasmid was introduced into the SA-2 strain, and the strains were cultivated in CGXII medium containing 50 g/L of glucose. Figure 2B shows shikimate production after 72 h of cultivation. SA-2 overexpressing *aroG* gene (SA-2/aroG) produced 5.4 ± 0.65 g/L of shikimate, 2.8-times higher than SA-2 (Figure 2A). Overexpression of aroB (SA-2/aroB) also slightly increased shikimate production (2.3 ± 0.23 g/L after 72 h). Overexpression of *qsuC, aroE1*, and *aroE3* did not increase shikimic acid production.

### Construction of *C. glutamicum* Strains With Integrated Shikimate Pathway Genes, and Introduction of Additional Shikimate Pathway Genes Using Plasmid-Based Expression

**FIGURE 3:**
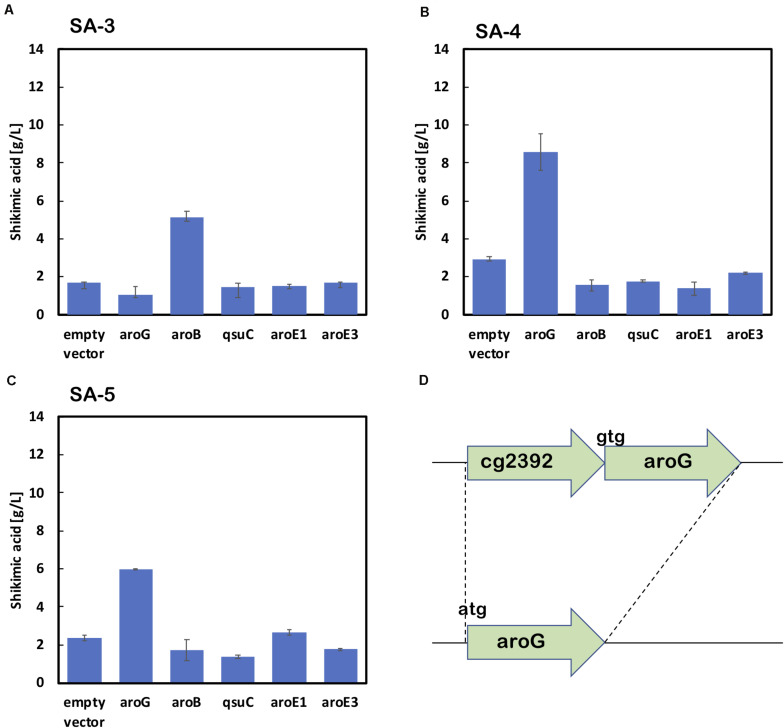
Shikimic acid production titer in strains **(A)** SA-3, **(B)** SA-4, and **(C)** SA-5 harboring each shikimate pathway gene expression plasmid. CGXII medium containing 50 g/L of glucose was used as the sole carbon source. Panel **(D)** illustrates a gene cg2392 deletion and replacement of the start codon in the SA-5 strain. The data are presented as the average of three independent experiments, and error bars indicate the standard deviation.

As shown in Figure 2B, the enhancement of *aroG* or *aroB* gene expression contributed to the increased production of shikimic acid. We therefore integrated an extra copy of *aroG* or *aroB* under the control of the promoter P_H__36_ into the *aroK* locus of the SA-2 genome. The resulting strains were named SA-3 (SA-2/aroK:P_H__36_-aroG) and SA-4 (SA-2/aroK:P_H__36_-aroB), respectively. To enhance the expression of *aroG*, we changed the start codon of *aroG* from “gtg” to “atg” (aroG^gtg→atg^) with an in-frame deletion of *cg2392* (NCgl2099) in SA-2 (Figure 3D). The resulting strain was named SA-5 (SA-2/Δcg2392, aroG^gtg→atg^). Changing the translational start codon of the gene increased its expression (Vogt et al., 2016; Jorge et al., 2017). The *aroG* gene is located downstream of *cg2392*, a predicted exonuclease, and they constitute an operon. In-frame deletion of *cg2392* and replacement of *aroG* downstream from the native promoter should increase *aroG* expression. To evaluate the shikimic acid production of SA-3, SA-4, and SA-5, we introduced an empty plasmid and cultivated the strains. Figures 3A–C (left bars of each panel) shows the shikimic acid production of SA-3, SA-4, and SA-5. Using CGXII medium containing 50 g/L of glucose, SA-3, SA-4, and SA-5 strains produced 1.7 ± 0.30 g/L, 3.0 ± 0.09 g/L, and 2.4 ± 0.15 g/L of shikimic acid, respectively. Statistical analysis revealed that significant difference was observed between SA-2 and SA-5, SA-3, and SA-5 (Supporting Figure S1). Glucose was consumed all strains, and the yield were 0.035 mol/mol (SA-3), 0.062 mol/mol (SA-4), 0.03550 mol/mol (SA-5), respectively. Unlike plasmid-based expression systems (Figure 2B), the integration of aroB increased shikimic acid production 1.5-fold over SA-2. In contrast, the integration of *aroG* into strain SA-2 decreased the production of shikimic acid. The SA-5 strain with the modified start codon had increased shikimic acid production by 26% compared with SA-2. These results suggest that *aroG* expression from the integrated *aroG* expression cassette was insufficient for shikimic acid production. In the case of *aroB*, a single integration of the *aroB* gene increased the shikimic acid titer over that of the plasmid-based overexpression system (SA-2/aroB; Figure 2B). Although we used medium copy number plasmid (about 30; Tauch, 2005), these results suggest that the production of large amounts of shikimic acid seems to depend on the plasmid copy number (Hashiro et al., 2019).

Using SA-3, SA-4, and SA-5 as host cells, each shikimate pathway gene *aroG*, *aroB*, *qsuD*, *aroE1*, and *aroE3* were introduced using a plasmid-based expression system. Figures 3A–C shows shikimic acid production after 72 h of cultivation. In the case of SA-3 (an *aroG*-integrated strain), additional plasmid-based *aroB* gene overexpression (SA-3/aroB) produced 5.1 ± 0.65 g/L of shikimic acid production, three-fold higher than SA-3. In strain SA-4 (an *aroB*-integrated strain), additional overexpression of *aroG* (SA-4/aroG) resulted in the highest shikimic acid production, up to 8.6 ± 0.96 g/L in CGXII minimal media containing 50 g/L of glucose. Overexpression of other genes did not improve shikimate production in either SA-3 or SA-4. The SA-5 strain expressing *aroG* (SA-5/aroG) increased the shikimate titer up to 6.0 ± 0.05 g/L, slightly higher than that of SA-2/aroG (5.4 ± 0.65 g/L; Figure 2B).

### Construction of *C. glutamicum* Strains With Integrated *aroG* and *aroB* Genes and Cultivation in Nutrient-Rich Media

**FIGURE 4:**
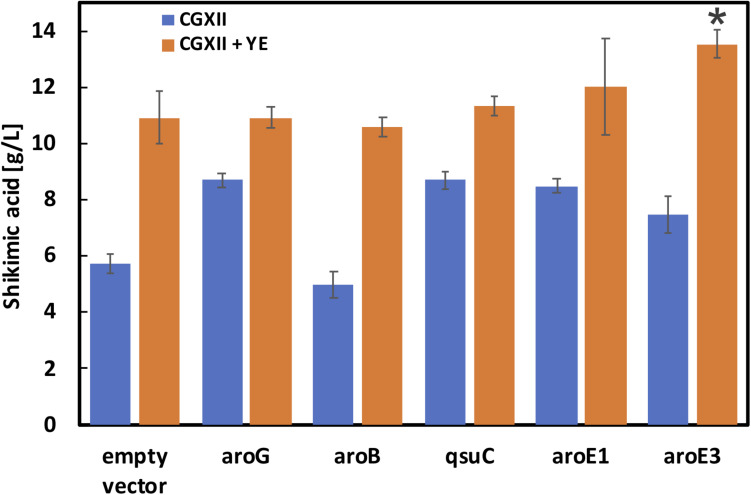
Shikimic acid production titer in SA-7 strains harboring each shikimate pathway gene expression plasmid. Blue bars indicate SA production when the CGXII medium containing 50 g/L of glucose was used as the sole carbon source. Orange bars indicate the CGXIIY medium used. The data are presented as the average of three independent experiments, and error bars indicate the standard deviation. *P* values were computed using the two-tailed Student’s *t*-test (*P < 0.05; **P < 0.01).

As shown in Figure 3, *aroG* and *aroB* are key genes for high shikimic acid production. Zhang et al. reported that high-level expression of aroG and medium-level expression of aroB using plasmids having different ribosome binding sites were suitable for shikimic acid production (Zhang et al., 2015b). In the engineered strain series developed by Kogure’s group, all of the genes of the shikimate pathway were expressed using a plasmid (Kogure et al., 2016). In this study, we introduced an *aroB* expression cassette into the *aroK* locus of the SA-5 strain. The resulting SA-6 strain produced 3.3 ± 0.29 g/L of shikimic acid (data not shown), which was slightly higher than the production of SA-5. We constructed strain SA-7 (SA-2/Δ*pta:aroG*, Δ*aroK:aroB*, Δ*cg2392*, *aroG*^*gtg*→*atg*^) by introducing an extra copy of the *aroG* gene chromosomally into the *pta* region of SA-6. The *pta* locus, encoding phosphate acetyltransferase, was selected for *aroG* integration to minimize any tendency for the formation of acetate as a byproduct. After cultivation, the SA-7 strain produced 5.7 ± 0.36 g/L of shikimic acid in CGXII medium containing 50 g/L of glucose (Figure 4, left blue bar), which was three-fold higher than SA-3 and 1.9-fold higher than SA-4. We additionally introduced each shikimate pathway gene into SA-7. SA-7 overexpressing *aroG* (SA-7/aroG) produced 8.7 ± 0.23 g/L of shikimic acid (Figure 4), almost the same as SA-4/aroG. SA-7 overexpressing *aroB* (SA-7/aroB) produced 5.0 ± 0.46 g/L of shikimic acid (Figure 4), almost the same as SA-4/aroB. These results suggest that a combination of plasmid-based aroG expression and single integration of *aroB* are valuable for high shikimic acid production. We measured the shikimic acid production of SA-7 transformants in nutrient-rich CGXIIY medium (CGXII medium containing 4 g/L of yeast extract and 50 g/L of glucose). Strain SA-7 carrying an empty plasmid produced 10.9 ± 0.92 g/L of shikimic acid (Figure 4, orange bar) with a yield of 0.22 g shikimate/g-glucose, about two-fold higher than SA-7 cultivated in CGXII minimal medium. Graf et al. reported that ATP and NADPH generation was enhanced in nutrient-rich medium (Graf et al., 2018), contributing to increased shikimic acid production. SA-7 overexpressing *aroG* and *aroB* strains produced 10.9 ± 0.38 g/L and 10.6 ± 0.36 g/L of shikimic acid in CGXIIY medium (Figure 4, orange bars), respectively, almost the same as SA-7 carrying an empty vector. Alternatively, SA-7 carrying plasmids containing *qsuC*, *aroE1*, or *aroE3* had slightly increased shikimic acid titers of up to 11.3 ± 0.31, 12.0 ± 1.70, and 13.5 ± 0.50 g/L, respectively. The one-way ANOVA analysis on the titer of shikimic acid production in CGXIIY medium revealed a statistically significant between empty vector and aroE3. These results indicate *aroE3* would be a rate-limiting step.

Feedback inhibition is another concern for shikimic acid production. In *E. coli*, AroG is specifically feedback regulated by L-phenylalanine, AroF and AroH are feedback regulated by L-tyrosine and L-tryptophan, respectively (Martinez et al., 2015). In the case of *C. glutamicum*, AroG activity was decreased only a 10 to 15% under 5 mM of L-phenylalanine and L-tyrosine concentrations (Liu et al., 2008). We also evaluated shikimic acid production CGXIIY medium including 100-fold higher (10 g/L) concentration of L-phenylalanine, L-tyrosine and L-tryptophan. The shikimic acid titer after 72 h was 6.6 ± 0.12 g/L (data not shown), about 40% decrease. These results suggest that the feedback inhibition of shikimic acid production with 100 mg/L (about 5–6 mM) of these amino acids was a small impact.

**FIGURE 5:**
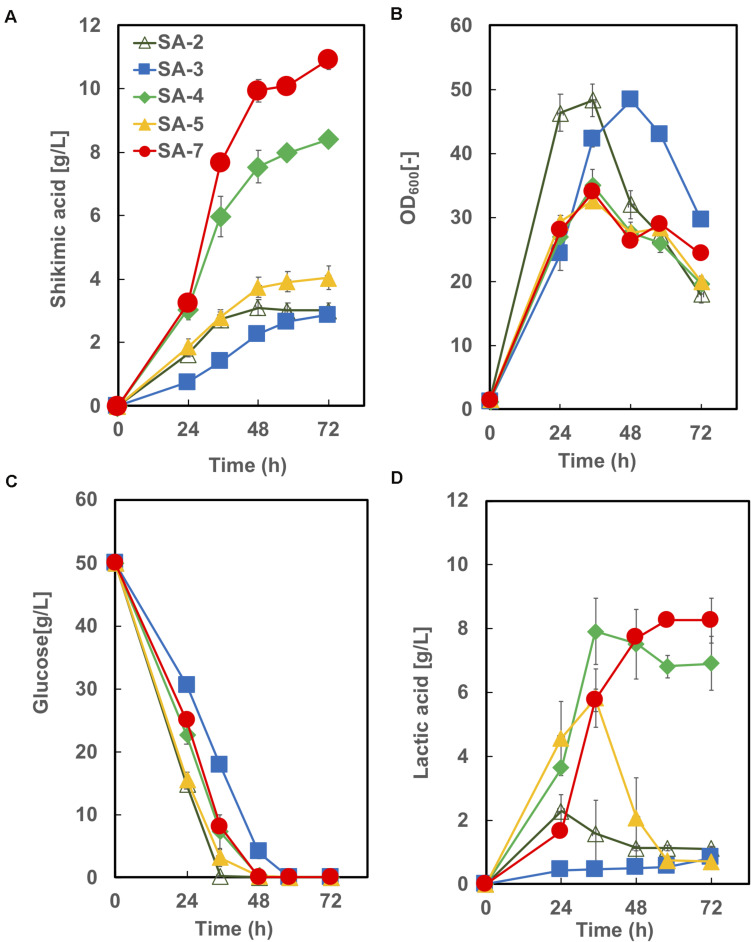
Culture profiles of SA-7 and its derived strains. **(A)** Shikimic acid production, **(B)** cell growth, **(C)** glucose consumption, and **(D)** lactic acid accumulation. The data are presented as the average of three independent experiments, and error bars indicate the standard deviation.

The time courses of each strain carrying an empty vector in CGXIIY medium containing 50 g/L of glucose are shown in Figure 5. SA-7 produced the highest amount of shikimic acid (10.9 ± 0.92 g/L), followed by SA-4, with 8.4 ± 0.19 g/L. The amount of shikimic acid produced by SA-4 in nutrient-rich CGXIIY medium was 2.8-fold higher than in CGXII minimum medium (Figure 3B, left bar). SA-2, SA-3, and SA-4 produced shikimic acid at 2.9 ± 0.24 g/L, 2.9 ± 0.04 g/L, and 4.0 ± 0.36 g/L, respectively, after 72 h in CGXIIY medium. The cell growth of SA-2 and SA-3 was higher than that of SA-7 and SA-4, suggesting that the carbon flux in SA-2 and SA-3 is distributed toward cell growth rather than shikimic acid synthesis. All strains except SA-3 consumed glucose for 48 h. Lactic acid was observed to be a major byproduct (Figure 4D). The SA-4 and SA-7 strains accumulated lactic acid at 6.9 ± 0.85 g/L and 8.3 ± 0.70 g/L, respectively. When these strains were cultivated in the CGXIIY medium without glucose, shikimic acid production was less than 0.5 g/L (data not shown). Acetic acid was not detected during cultivation in SA-7 or other strains. 3-dehydroshikimate, reported as a major precursor, was not detected in any strain.

### Evaluation of Cofactor Balance and Transcription Levels of the Lactate Dehydrogenase Gene in SA-6

**FIGURE 6:**
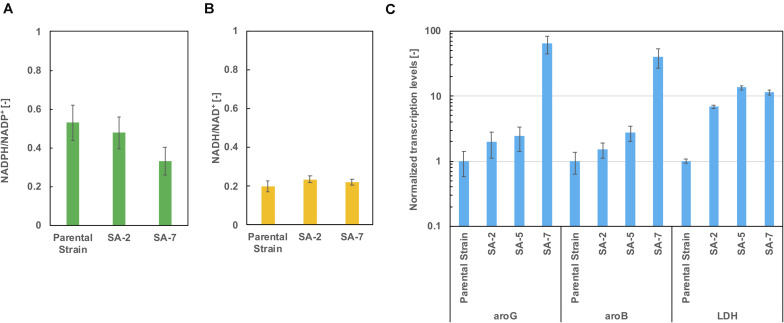
The ratio of **(A)** NADPH/NADP + and **(B)** NADH/NAD + after 24 h cultivation in CGXIIY medium. Panel **(C)** shows the transcription levels of *aroG*, *aroB*, and *ldh* after 24 h of cultivation. The data are presented as the average of three independent experiments, and error bars indicate the standard deviation.

As shown in Figure 5D, lactic acid accumulation was a major metabolic byproduct. Figures 6A,B show intracellular NADPH/NADP + and NADH/NAD + ratios after 24 h of cultivation in CGXIIY medium. In spite of lactic acid formation, as shown in Figure 5D, the NADH/NAD + ratio was almost the same among the parental strains SA-2 and SA-7. The NADPH/NADP + ratio of SA-7 was decreased relative to SA-2 due to the final reaction of shikimic acid synthesis, which is catalyzed by shikimate dehydrogenase, *aroE*. These results are consistent with previous reports (Kogure et al., 2016).

The transcription levels of *aroG*, *aroB*, and lactate dehydrogenase gene *ldh* were evaluated using real-time qPCR after 24 h of cultivation. The *aroG* levels of the SA-5 strain were slightly increased compared with those of SA-2 (Figure 6C), suggesting that a gene *cg2391* deletion had little influence on aroG transcription level (Figure 4D). Alternatively, in the case of the SA-7 strain, in which *aroG* and *aroB* are integrated under the control of the P_*H*__36_ promoter, the transcription levels of *aroG* and *aroB* were significantly increased, up to 64.3- and 40.0-fold higher than those of the parental strain, contributing to improved shikimic acid production. The transcription levels of *ldh* were increased in all strains other than the parental strain. We disrupted the *ldh* gene (cg3219, NCgl2810) in SA-7. The resulting strain, SA-8, produced 9.9 ± 0.78 g/L of shikimic acid and accumulated 8.1 ± 0.18 g/L of lactic acid (Supplementary Figure S2), almost the same as SA-7. *C. glutamicum* has another putative lactate dehydrogenase gene (cg3227, NCgl2817), which might contribute lacate accumulation. A gene *lldD* (cg3227, NCgl2817) encoding quinone-dependent L-lactate dehydrogenase has the ability to utilize lactate as a carbon source (Stansen et al., 2005), however, activation of lactate re-assimilation pathway required a full oxygen supply (Käß et al., 2014). A limited-oxygen condition after glucose depletion in the test tube was another factor for lactate accumulation.

### Construction of *C. glutamicum* Strains With Integrated *aroG*, *aroB*, and *aroE3* Genes and Shikimic Acid Production Using Cellobiose as a Carbon Source

**FIGURE 7:**
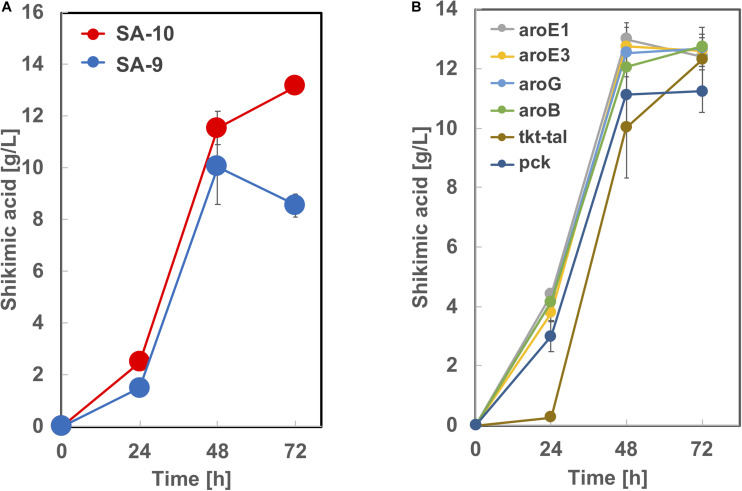
Shikimic acid production titer in **(A)** SA-9, SA-10 and **(B)** its derived strains. CGXII medium containing 50 g/L of glucose was used as the sole carbon source. The data are presented as the average of three independent experiments, and error bars indicate the standard deviation.

Based on the results from Figure 5, we constructed strain SA-9 (SA-7/Δ*ldh:aroE1*) and SA-10 (SA-7/Δ*ldh:aroE3)* by introducing an extra copy of the *aroE1* or *aroE3* gene chromosomally into the *ldh* region of SA-7, respectively. After 72 h cultivation, the SA-9 strain produced 8.5 ± 0.46 g/L of shikimic acid in CGXIIY medium containing 50 g/L of glucose (Figure 7A). Alternatively, SA-10 produced 13.1 ± 0.25 g/L of shikimic acid with the yield of 0.26 g-shikimic acid/g-glucose (Figure 7A), which was almost same level of SA-7 harboring aroE3 expressing plasmid (Figure 4). We then focused on improving the availability of the shikimate pathway precursors PEP and E4P. To increase the supply of E4P, we constructed a plasmid for the overexpression of the endogenous transketolase (*tkt*) and transaldolase (*tal*) genes (cg1774-cg1776, NCgl1512-NCgl1513) and introduced into SA-10. Alternatively, to increase the availability of PEP, a plasmid for overexpression of endogenous phosphoenolpyruvate carboxykinase gene (*pck*; cg3169, NCgl2765) was introduced into SA-10. The resulting strain produced shikimic acid at 11.2 ± 0.71 g/L and 12.3 ± 0.25 g/L, respectively (Figure 7B). We also introduced *aroB*, *aroG*, *aroE1*, and *aroE3* expression plasmid into SA-10 strain. Each strain produced shikimic acid at 12.8 ± 0.11 g/L, 12.7 ± 0.37 g/L, 12.4 ± 0.34 g/L and 12.6 ± 0.55 g/L, respectively (Figure 7B).

**FIGURE 8:**
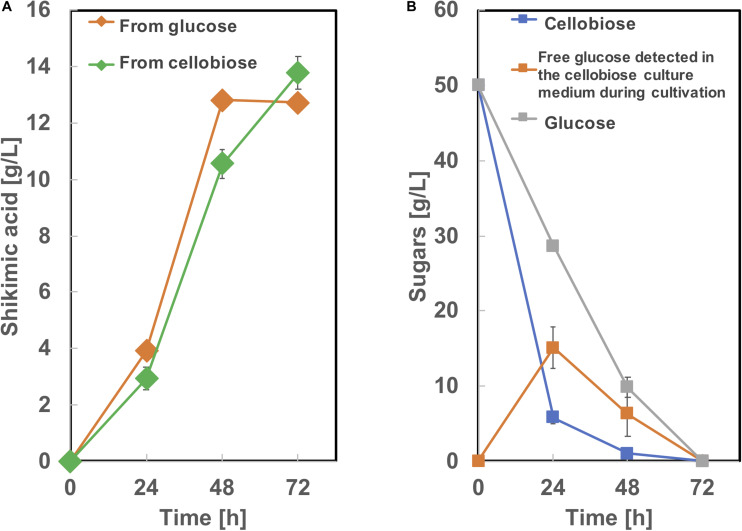
Culture profiles of BGL expressing SA-10 strain. **(A)** Shikimic acid production, **(B)** cellobiose and glucose consumption. The data are presented as the average of three independent experiments, and error bars indicate the standard deviation.

To produce shikimic acid from cellobiose as a carbon source, we used BGL Tfu0937 from *Thermobifida fusca* YX, which has been shown to exhibit high BGL activity in *E. coli* (Tanaka et al., 2011) and *C. glutamicum* (Matsuura et al., 2019). A plasmid secretory expressing BGL (Matsuura et al., 2019) was introduced into SA-10 and cultivated in CGXIIY medium containing 50 g/L of glucose or cellobiose as a carbon source. As shown in Figure 8, this BGL-secreting SA-10 strain produced 13.8 ± 0.56 g/L of shikimic acid from cellobiose as well as 12.7 ± 0.19 g/L of shikimic acid from glucose. The yield from cellobiose is 0.25 g-shikimic acid/g-glucose (1 g of cellobiose corresponds to 1.1 g of glucose), which was almost same as that from glucose (0.25 g-shikimic acid/g-glucose). These results show BGL expression did not affect shikimic acid production, which are corresponding to previous report (Matsuura et al., 2019). Cellobiose was completely consumed by 72h. However, free glucose was observed in the culture medium at 24 and 48 h cultivation. indicating the conversion of cellobiose into glucose by BGL was sufficient and improvement of glucose uptake might enhance shikimic acid production.

Microbial shikimic acid production has been reported by many groups. An engineered *E. coli* strain reported by Chandran et al. (2003) produced 87 g/L of shikimic acid from glucose with a yield of 0.36 (mol/mol) in fed-batch fermentation. Rodriguez et al. (2013) reported a metabolically engineered *E. coli* strain and the strain produced 43 g/L of shikimic acid with the yield of 0.43 (mol/mol) in batch culture. In the case of *C. glutamicum*, Kogure et al. (2016) reported 141 g/L of shikimic acid production using growth-arrested cell reaction with the yield of 0.51 (mol/mol). In our study, although shikimic acid titer (13.1 g/L) and yield (0.27 mol/mol) were less than those reports, we developed high shikimic acid producing *C. glutamicum* ATCC13032 strain without loss of PTS function and pyk activity. It should be useful property for the synthesis of plant polyphenols. In addition, we demonstrated direct shikimic acid production from cellobiose, which has the possibility of the reduction of the production cost.

## Conclusion

In conclusion, The *C. glutamicum* strain SA-10 was metabolically engineered for enhanced shikimic acid production from glucose by optimizing its metabolic pathway, retaining the PTS system and *pyk* activity. Genomic integration of the *aroB, aroG* and *aroE3* genes improved shikimic acid production up to 13.1 g/L with a yield of 0.26 g-shikimic acid/g-glucose. Moreover, BGL-expressing SA-10 strain produced shikimic acid from cellobiose with almost same titer and yield from glucose. This study is an important step in developing an economically feasible and sustainable process for shikimic acid production.

## Data Availability Statement

The datasets presented in this study can be found in online repositories. The names of the repository/repositories and accession number(s) can be found in the article/ Supplementary Material.

## Author Contributions

NS and TT proposed the idea, designed the experiments, and wrote the manuscript. NS, MK, MN, YH, and TT performed the experiments. All authors read and approved the final manuscript.

## Conflict of Interest

The authors declare that the research was conducted in the absence of any commercial or financial relationships that could be construed as a potential conflict of interest.

## Supplementary Material

FIGURE S1 Culture profiles of strain SA-2, SA-3 and SA-5. The data are presented as the average of three independent experiments, and error bars indicate the standard deviation. *P* values were computed using the two-tailed Student’s *t*-test (^∗^, *P* < 0.05).

FIGURE S2 Culture profiles of strain SA-8. **(A)** Shikimic acid production, **(B)** cell growth, **(C)** glucose consumption, **(D)** lactic acid accumulation. The data are presented as the average of three independent experiments, and error bars indicate the standard deviation.

TABLE S1 Oligonucleotide primers used in this study.
