# Supplementary material for: Metabolic Engineering of Shikimic Acid-Producing Corynebacterium glutamicum From Glucose and Cellobiose Retaining Its Phosphotransferase System Function and Pyruvate Kinase Activities
Source: Front Bioeng Biotechnol. 2020 Sep 10;8:569406. doi: 10.3389/fbioe.2020.569406 (PMC7511668; doi:10.3389/fbioe.2020.569406)
Supplement: FIGURE S1 — Culture profiles of strain SA-2, SA-3 and SA-5. The data are presented as the average of three independent experiments, and error bars indicate the standard deviation. P values were computed using the two-tailed Student’s t-test (∗, P < 0.05). [file Data_Sheet_1.pdf]

Supplementary Material

1 Supplementary Data

1.1 Supplementary Figure

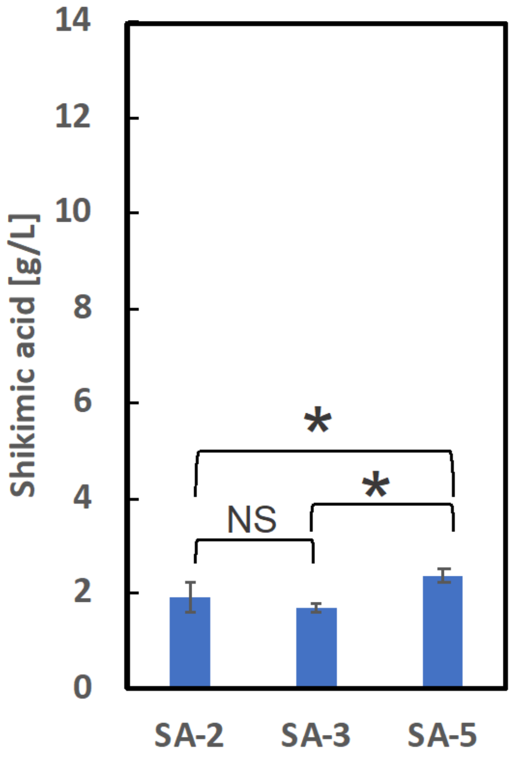

Supplementary Figure 1. Culture profiles of strain SA-2, SA-3 and SA-5. The data are presented as the average of three independent experiments, and error bars indicate the standard deviation. P values were computed using the two-tailed Student's t-test (*, $P < 0.05$).

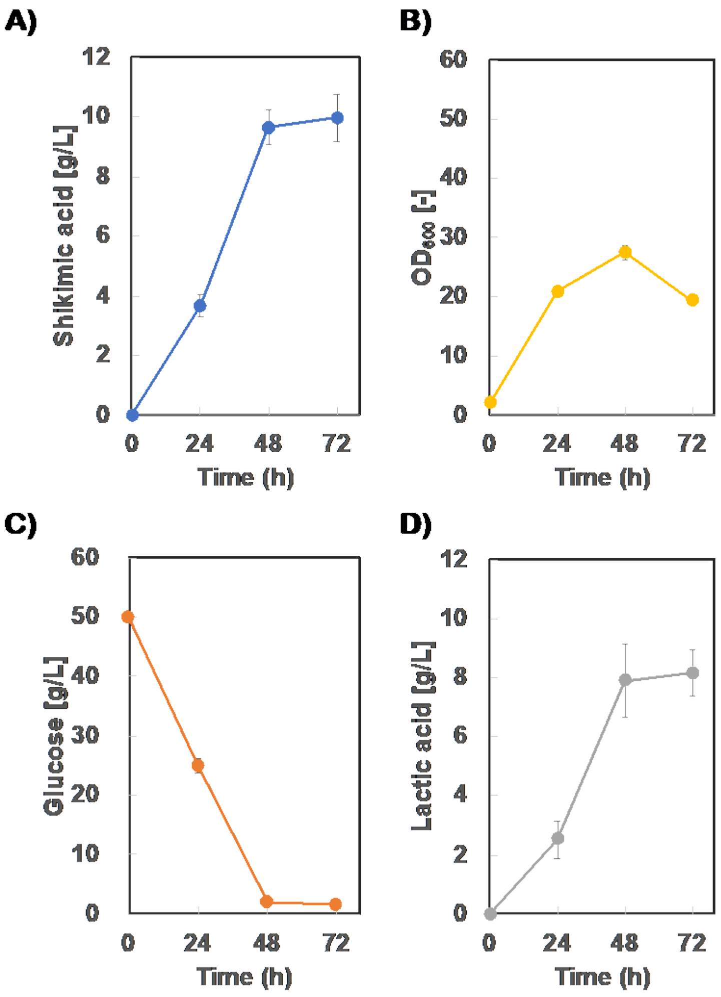

Supplementary Figure 2. Culture profiles of strain SA-8. (A) Shikimic acid production, (B) cell growth, (C) glucose consumption, (D) lactic acid accumulation. The data are presented as the average of three independent experiments, and error bars indicate the standard deviation.

2 Supplementary table

2.1 Supplementary Table S1 Oligonucleotide primers used in this study.

Oligonucleotide primers	Sequence
ΔaroK_up_fw	5'- CATGATTACGAATTCTTCGATGGACTACAG -3'
ΔaroK_up_rv	5'- GGTTTAATTAATCGCCTCTAAACCTTC -3'
ΔaroK_down_fw	5'- GAAGGTTTAGAGGCGATTAATTAAACC -3'
ΔaroK_down_rv	5'- AGTGCCAAGCTTTGGCTGATTGCC -3'
ΔqsuD_up_fw	5'- CATGATTACGAATTCTGGCGGTGGCTGAA -3'
ΔqsuD_up_rv	5'- CGAGGTTTTACTGACTCCTACTTTTTGAGATTTG -3'
ΔqsuD_down_fw	5'- CAAATCTCAAAAAGTAGGAGTCAGTAAAACCTCG -3'

ΔqsuD_down_rv	5'- GACTCTAGAGGATCCCATCACTTGCTCCCT -3'
ΔqsuB_up_fw	5'- ACATGATTACGAATTCCAGTGTTCATCATGGGTGCCATTG -3'
ΔqsuB_up_rv	5'- CACGATGCAACTGCTCTTTGGTTTTACTGAGCCGCGAGTTG -3'
ΔqsuB_down_fw	5'- CGGCTCAGTAAAACCAAAGAGCAGTTGCATCGTGCAGCAG -3'
ΔqsuB_down_rv	5'- CGACTCTAGAGGATCCCAATGAAATCAACACCCTCAGGTTCCG -3'
ΔnagD_up_fw	5'- ACATGATTACGAATTCGTCGGAGTCCACGATGGTGTGG -3'
ΔnagD_up_rv	5'- GCTGCTCATCTGGTACGTGCACCATTGTCCCTGTTTTGGGC -3'
ΔnagD_down_fw	5'- CAGGGACAATGGTGCACGTACCAGATGAGCAGCAGTTCAC -3'
ΔnagD_down_rv	5'- CGACTCTAGAGGATCCGTGAACTGCTGCTCATCTGGTACGTG -3'
gnd_S361F_up_fw	5'- CTATGACATGATTACGAATTCGTCCATCGCTGCCAA -3'

gnd_S361F_up_rv	5’- GGGTCGACGTCCCAGTTGTTCTCGTCAAAGCCAGCCTTGATCTCGTC -3’
gnd_S361F_down_fw	5’- GCTTTGACGAGAACAACTGGGACGTCGACCCTCGCGACCTCGCTAC -3’
gnd_S361F_down_rv	5’- CGACTCTAGAGGATCCCCGGGTTAAGCTTCAACCT -3’
aroK_up_fw	5’- CATGATTACGAATTCGTAATTTCCATTGGT -3’
aroK_up_rv	5’- CCAGATAGAGAATTCCCTCTAAACCTTCGA -3’
aroK_down_fw	5’- TCTGTTGTTCTCGACCGATTAATTAAACCG -3’
aroK_down_rv	5’- AGACTCTAGAGGATCCGATCAGTTTCGTAA-3’
P_aroG_T_fw	5’- TCGAAGGTTTAGAGGGAATTCTCTATCTGG -3’
P_aroG_T_rv	5’- CGGTTTAATTAATCGGTCGAGAACAACAGA -3’
Δcg2392_up_fw	5’- ACATGATTACGAATTCCTTGAGCACTCGGCGCAAGACGATAG -3’

Δcg2392_up_rv	5’- GGGATATCAACTGTCCAACTCATTAGCAATTAGTCCTTCACAG CGTCGAAAACG -3’
Δcg2392_down_fw	5’- CGCTGTGAAGGACTAATTGCTAATGAGTTGGACAGTTGATATCCCTAAAGAAGTTCTCCC -3’
Δcg2392_down_rv	5’- CGACTCTAGAGGATCCCTCGCTGAGACGGTAAAGATCAGCGGTG -3’
pta_up_fw	5’- ACATGATTACGAATTCAATGTGGAGTATGTGAGCGGGGGTTC -3’
pta_up_rv	5’- GCTGCTGGCGGCCGCCAGCCTGAACCTTCTCATTGATGACACTTCAG -3’
pta_down_fw	5’- GGTTCAGGCTGGCGGCCGCCAGCAGCATTTACCGCTGAACAAGAGCC -3’
pta_down_rv	5’- CGACTCTAGAGGATCCGTTGGGTTCGGGTTAACAGCACAGTC -3’
ldh_up_fw	5’- ACATGATTACGAATTCCCGATAAAGCCAGCGCCCATATTT -3’
ldh_up_rv	5’- GATTTCATAATCTTGACGCGGCCGCTTTCGATCCCACTTCCTGATTTCCC -3’

ldh_down_fw	5'- GATCGAAAGCGGCCGCGTCAAGATTATGAAATCCATCGTCGGCGATG -3'
ldh_down_rv	5'- CGACTCTAGAGGATCCGCGATGTCTTCCTCACCGTATTCACCGTG -3'
aroK-H36_fw	5'- GAATTCTCTATCTGGTGCCCTAAACGGGGG -3'
aroK-term_re	5'- GTCGAGAACAACAGATAAAACGAAAGGCCC -3'
pta-H36_fw	5'- AAGGTTCAGGCTGGCGGCCGCCCATTCAGGCTGCGCAACTGTTG -3'
pta-term_re	5'- ATGCTGCTGGCGGCCGCTGTTATGTGCCTGGCTCCTAATCAAAGCTG -3'
ldh-H36_fw	5'- AGTGGGATCGAAAGCGGCCGCCCATTCAGGCTGCGCAACTGTTG -3'
ldh-term_re	5'- AATCTTGACGCGGCCGCTGTTATGTGCCTGGCTCCTAATCAAAGCTG -3'
aroG_fw	5'- GAGTAGCATGGGATCCATGAGTTGGACAGTTGATATCCCTAAAGAAGTTCTCCCTG -3'
aroG_rv	5'- ACAGCCAAGCCTCGAGTTAGTTACGCAGCATTTCTGCAACGAGGAAAGC -3'

aroB_fw	5’- GAGTAGCATGGGATCCATGAGCGCAGTGCAGATTTTCAACACCGTC -3’
aroB_rv	5’- ACAGCCAAGCCTCGAGTTAGTGGCTGATTGCCTCATAAGCACTCTGTAAATCTTG -3’
qsuC_fw	5’- TAGGAGTAGCATGGGATCCATGCCTGGAAAAATT -3’
qsuC_rv	5’- CAAAACAGCCAAGCCTCGAGCTACTTTTTGAGATT -3’
aroE1_fw	5’- GAGTAGCATGGGATCCATGGTCAACTACGTCGACAGGGAAACAACCCTG -3’
aroE1_rv	5’- ACAGCCAAGCCTCGAGTTACTTGGAGAACTCCTCCGCAGCAATGATCTG -3’
aroE3_fw	5’- GAGTAGCATGGGATCCATGGGTTCTCACATCACTCACCGGGC -3’
aroE3_rv	5’- ACAGCCAAGCCTCGAGTTAGTGTTCTTCTGAGATGCCTAAAGACTCTTCCAAAGCCTC -3’
tkt-tal_fw	5’- GAGTAGCATGGGATCCATGACCACCTTGACGCTGTCACCTG -3’
tkt-tal_rv	5’- ACAGCCAAGCCTCGAGCTACTTCAGGCGAGCTTCCATGGACTCAAG-3’

pck_fw	5’- GAGTAGCATGGGATCCATGACTACTGCTGCAATCAGGGGCCTTC-3’
pck_rv	5’- ACAGCCAAGCCTCGAGTTAAGCGTGAGCTGCTGAAATGCGG-3’
RT-aroG_fw	5’- TTGCATACGCTGACAAGCTC -3’
RT-aroG_rv	5’- TGCCTCAACAGCCTGGATAA -3’
RT-aroB_fw	5’- CTGCGCCTTTACGAAACTGA -3’
RT-aroB_rv	5’- TGGGAGCCTTCGACTTCTTT -3’
RT-ldh_fw	5’- GCATACGCATACGCACTGAT -3’
RT-ldh_rv	5’- CATGACGTTGCCTTCGAGTT -3’
RT-cg3177_fw	5’- GCATACGCATACGCACTGAT -3’
RT-cg3177_rv	5’- CATGACGTTGCCTTCGAGTT -3’
